# Laughter

**Published:** 1883-01

**Authors:** Ausburn Towner


### LAUGHTER.

AUSBURN TOWNER.

The capacity of laughing belongs with sleep, appetite and taste, among the best gifts to man. It deserves to stand at the head of the various attributes of the human species too, because we hold in common with all animate creation, our faculties and capacities, this one being the only exception. It especially belongs to the human race, and is so entirely distinct, peculiar and different from any act performed by any other species of animals; it is such a bar between man and the lower orders of creation, that it forms an argument against the notion of evolution, impossible for Darwin and his followers to climb over. The first man must have been as able to laugh heartily as is the latest born child. It is something that could not have been developed, evolved or grown up to. The first diaphragm or midriff must have been as sensitive and elastic as is the latest, to respond to the emotions excited by mirth or other pleasant reflections.

I am disposed to disagree with the commonly accepted notion that there are various kinds of laughter, and the origin or etymology of the word bears me out in this. Its true meaning involves the notion of mirth, merriment, joy, gladness and exhilaration, and to mix with it any sense of scorn, frivolity, derision or other uncomfortable sentiment, entirely destroys its character, beauty or usefulness. Where these latter, or ideas akin to them are expressed, it may be that the same organs of the body are used and in a similar manner as where there is genuine laughter, but it is no more like than is eating when you are not hungry or drinking when you are not dry, to eating with a vigorous appetite or drinking to quench thirst. What is more uncomfortable to execute or observe than a forced laugh? In all of these instances, the same organs are used and the form is gone through with, but no satisfaction follows, if positive injury is not the result.

I think the poverty of the early English language is shown in the use of the term laughter, where something else is intended, like a sneer or scorn. In the Bible, for instance, the word is never used, with one exception, in the true sense connecting it with gladness and good feeling. In the well-known quotation from Job: "When the morning stars sang together and all the sons of God *shouted* for joy," how much better it would have been if the word "laughed" could have been used in the translation, instead of the one in italics, and I am of the impression that that was the sense intended in the original. Certainly to laugh for joy would be more expressive than to shout, and quite as natural. Where there are mentions made of other personages laughing, the term means something else, except perhaps in the case of Sarah, the one exception alluded to above, who, when she was well stricken in age, on hearing one of the three angels say that she should bear a son, laughed. She might have done so, either because she thought it a good joke or because she was pleased that such an event was possible.

The other laughing of the Bible has to do with scorn or calamity. "At destruction and famine thou shalt laugh;" "I also will laugh at your calamity;" "All they that see me laugh me to scorn," and the like. Our own consciousness tells us every time that some other term should have been used, for it is as inaccurate as another expression of Job, in relation to Leviathan: "He laugheth at the shaking of a spear."

Although in the Bible proper, the term is so misapplied, there is one curious instance in the Apocrypha, where an account is given of some genuine laughter. In the "History of Bel and the Dragon," the King rebukes Daniel for not worshiping the idol. "Thinkest thou not that Bel is a living God?" the King asked. "Seest thou not how much he eateth and drinketh every day?" "Then," says the History, "Daniel smiled." He knew that the priests themselves, and not the idol ate up the fine flour and fat sheep and drank the wine provided for the temple. Further along, after Daniel had fixed up his trap for the priests and had caught them in it, he laughed, and with good reason. This was genuine laughter, founded on what was fun for Daniel.

It is a somewhat singular fact that, although we are told our Lord wept, we never hear that He laughed, or even smiled. A French philosopher, who contended that laughter was caused by novelty or surprise, suggested that the cause of the above fact, was that nothing was new to Him. Other more plausible and patent reasons suggest themselves, and indeed I know of no two words joined together that would strike harder on the sensibilities or seem more ill-connected than the two: "Jesus laughed," His whole character and life being so much more made up of tears, sorrow and grief, than gladness or joy. Still, what is more gracious or more indicative of His loving kindness, than the expression, "The smile of Jesus," and a smile is the skirmish line of a laugh.

The English language of to-day is largely indebted to the Bible for what it is, and it is more than probable that the intermingling of sentiment in the term "laughter," employed in that book, has given it its present complicated meaning, and thus somewhat degraded it. Besides, the somber cast of character of the ancient Jews, like that of the American Indians, may have made hearty, honest, genuine laughter impossible for them. When thinking of these people in this light, one is reminded of the incident of the little girl who was reading the history of England with her governess. They came to the statement that the great Henry I. never laughed again after the death of his son. The little girl stopped, looked up and asked: "What did he do then, when he was tickled?"

It is a reasonable subject for regret that our tongue has no term that expresses the real meaning implied by the word "laughter." You are obliged to qualify it to indicate what sense you would convey to your hearer or reader. "Received with shouts of laughter," may mean shouts of scorn, sneers, sympathy, dislike, gladness or merriment, and so really mean nothing. It is to be deplored that we have to use the same word to describe the merry and amused manner in which an innocent child expresses its emotions of pleasure, that we do to speak of the horrid shrieks of a maniac.

Besides the influence of the Bible in the construction of the language already alluded to, there are other causes that have combined to lower the act and the term in the estimation of mankind. This most exhilarating, health-giving and strengthening exercise of some of the most important portions of the human body has been deemed a device of the devil, a trap by which the unwary are sometimes caught! This was the Puritanic notion of laughter. But if you were to choose between two strangers which one to entrust with an important matter, would you take the one who never laughed, or the one whose quick perceptions caught hold of a point instantly and responded to it with an open, free and hearty laugh? Laughter has also been considered an evidence of ill-breeding. Lord Chesterfield disapproved of it on this account, and further, because the noise was disagreeable, and because of the "shocking distortion of the face that it occasioned!" Just as wise a man as Chesterfield, Lord Bolingbroke, said that "gravity is the very essence of imposture," and a wiser than either, has observed that "the gravest beast is the ass; the gravest bird is the owl; the gravest fish is the oyster, and the gravest man is a fool." Plato, the great philosopher, was once with his attendants, indulging in the gaiety of his heart, no doubt laughing unconstrainedly, when he suddenly stopped and smoothing down his face, exclaimed: "Silence, my friends! let us be wise now; here is a fool coming." Laughter has been found a weapon that can overcome any argument, and its good-natured character has been degraded by being put to such a use. It has also been called undignified, unmanly and childish. This was a notion of the stoics and of many of the religious orders, the value of whose opinions may be estimated from the fact that they endeavored to repress if not to destroy all of the gentler, kindlier and humane sentiments of the human heart. And as to dignity, where can be found more dignified characters in history than Philip of Macedon, and Sulla, the Roman general, both of whom delighted in jokes and laughed at them; or Julius Cæsar and Tacitus the historian, both of whom loved so well to laugh that they made a collection of jests and enjoyed them constantly, or Queen Elizabeth, of England, who could laugh at a joke or a smart saying, with the lightheartedness of any of her maids of honor.

It is unfortunate that all of these things have conspired to complicate the notion of laughter. It would have been much better if it could have retained its single meaning and not been confounded even with frivolity; when the expression, "the laughter of fools," would have been inconsistent, and such lines as that of Goldsmith, in the "Deserted Village," "And the loud laugh that spoke the vacant mind," could never have been written.

I do not pretend to say that what I define as laughter is by any means one of the most important duties of life, or that it can not be untimely or out of place; but I do claim that it deserves a higher place in our estimation than it has with many, and that those who can produce it in its purity and innocence without any alloy of malice or unkindness, merit the title of benefactors and philosophers, rather than clowns, apes or fools. If a man who can make two blades of grass grow where there was before but one, is entitled to immortality and blessings, one who can strike out a new thought, so surprising and taking that it will lift the diaphragm into a hearty laugh, earns for himself just as enviable a place. I say this with much reason too, for mankind has been laughing for ages at the same humorous stories. The famous rhyme:

"Mother may I go out to swim,
Yes my darling daughter,
Hang your clothes on a hickory limb
And don't go near the water,"

which no one can read or hear, for the first time, without laughing, is thirteen hundred years old we know, for it was in a book of jests issued in the sixth century, by Hierocles, and we don't know where he got it from. In the same book is the story of the man who complained that his horse died just as he had taught it to live without food, and of the one who meeting a friend, asked whether it was he or his brother who had been buried. That is a laughter provoking remark that appears now and then fresh and new, where a friend presents to a visitor some very old wine in a very small glass, and the visitor says as he receives it, that it is very little for its age. It was said twenty-two hundred years ago, and we do not know as then for the first time, by Phyrne, the Athenian courtesan, who was the model for the Venus of Praxiteles. Every one knows of the story and beauty of this woman. You have doubtless heard the end man at a minstrel show tell of seeing his friend in jail, and asking how he came there, gets the reply that it was for picking up an old rope in the street. That seems queer until it is explained that there was a cow tied to the other end of the rope. That story is in an old Chinese jest book, printed more than two thousand years ago. These are but examples that might be indefinitely extended, going to show how high are the merits of one who discovers a new source for the creation of laughter. The world, whatever it may think of or however it may treat these discoveries, speedily catches up the laugh and it echoes around the world. Dickens, in his Sam Weller, originated a species of laughter producing quibbles that were very enjoyable, and made him his first steps to immortality. While the Pickwick Papers were being published, every one was quoting a "Wellerism" like "Out with it, as the father said to the child, when he swallowed a farden," or making imitations of them, until they became tiresome. In our generation, much the same thing is being done by Stanley Huntley, in what he calls the "Spoopendyke Troubles." Mr. Huntley has not wisely chosen a name, and his lucubrations are unfortunately of such an ephemeral character as to be printed in a daily newspaper, a sepulchre for literary efforts at the best, but he has added a laughter provoking expression to the world that is not a whit behind a Wellerism. Here is one of them: "Oh, you've got it!" snorted Mr. Spoopendyke to his wife. "If you only had red cushions and a rack nailed up in front of you, you'd only need an overdue mortgage and a fight in the choir to be a dod-gasted fashionable church!" There is still another one who has added a new source of laughter to the world in the Chicago *Tribune*. He writes of the love passages between Gwendolen McCarthy and Bertie Cyril. These are not striking, but he has developed an old comparison most wonderfully. In speaking of a man with a benign expression of countenance, some one added: "Yes, a seven-by-nine one," a rather paneful, but oftentimes expressive extension of the first idea. The *Tribune* man has still further elongated this and speaks of a yearning, a "reaching-around-the-corner-and-up-two-pair-of-stairs-for your-spring-over-coat yearning," that makes one break out all over with laughter.

I would advise all who can laugh to do so, if they see or hear any thing that excites their risibilities. Let it come out free and hearty. It makes cares and responsibilities not only seem to be, but really lighter. And if you can not yourself laugh, either through some disarrangement of the organs near the diaphragm, or from some outside disagreement or disturbance, go where you can hear some one laugh. It has been said that to those in sorrow, a laugh is like a mockery. A good, genuine laugh may be untimely, but it can never be a mockery. A calamity or a misery may be treated with scorn or sneers, but no one could ever laugh at another's misfortune in the only real and honest sense that belongs to laughter. That is too genial and sympathising. It is balm to the wounded and medicine to the sick. I have seen pale and suffering men raise the curtains of their beds to hear more clearly the healthy laughter that came to their ears from an adjoining room, and take comfort in the sound, and I have seen the light of a beginning recovery leap to the eyes of one sick to death, at the familiar laugh of a loved physician, for whose coming he had waited patiently many days, rolling up to him from the hall beneath. Alas! for the man who can not laugh or who can not bear to hear others laugh. His life is a blank, and he has no distinguishing difference between himself and the beasts of the field.

